# Clinical Significances of Anti-Collagen Type I and Type III Antibodies in Antibody-Mediated Rejection

**DOI:** 10.3389/ti.2022.10099

**Published:** 2022-05-11

**Authors:** Sehoon Park, Seung-Hee Yang, Jiyeon Kim, Semin Cho, Jaeseok Yang, Sang-Il Min, Jongwon Ha, Chang Wook Jeong, Seong Hee Bhoo, Yong Chul Kim, Dong Ki Kim, Kook-Hwan Oh, Kwon Wook Joo, Yon Su Kim, Kyung Chul Moon, Eun Young Song, Hajeong Lee

**Affiliations:** ^1^ Department of Internal Medicine, Armed Forces Capital Hospital, Seongnam-si, South Korea; ^2^ Department of Biomedical Sciences, Seoul National University College of Medicine, Seoul, South Korea; ^3^ Kidney Research Institute, Seoul National University, Seoul, South Korea; ^4^ Department of Internal Medicine, Seoul National University Hospital, Seoul, South Korea; ^5^ Transplantation Center, Department of Surgery, Seoul National University Hospital, Seoul, South Korea; ^6^ Department of Surgery, Seoul National University College of Medicine, Seoul, South Korea; ^7^ Department of Urology, Seoul National University Hospital, Seoul, South Korea; ^8^ Department of Genetic Engineering and Graduate School of Biotechnology, Kyung Hee University, Yongin-si, South Korea; ^9^ Deparment of Internal Medicine, Seoul National University College of Medicine, Seoul, South Korea; ^10^ Department of Pathology, Seoul National University Hospital, Seoul, South Korea; ^11^ Department of Laboratory Medicine, Seoul National University Hospital, Seoul, South Korea

**Keywords:** kidney transplantation, kidney, non-HLA antibody, antibody-mediated rejection, graft failure

## Abstract

It is important to determine the clinical significance of non-human leukocyte antigen (HLA) antibodies and their association with antibody-mediated rejection (ABMR) of kidney allografts. We collected post-transplant sera from 68 ABMR patients, 67 T-cell mediated rejection (TCMR) patients, and 83 control subjects without rejection, and determined the titers of 39 non-HLA antibodies including antibodies for angiotensin II receptor type I and MICA. We compared all these non-HLA antibody titers among the study groups. Then, we investigated their association with the risk of death-censored graft failure in ABMR cases. Among the antibodies evaluated, anti-collagen type I (*p* = 0.001) and type III (*p* < 0.001) antibody titers were significantly higher in ABMR cases than in both TCMR cases and no-rejection controls. Both anti-collagen type I [per 1 standard deviation (SD), adjusted odds ratio (OR), 11.72 (2.73–76.30)] and type III [per 1 SD, adjusted OR, 6.22 (1.91–31.75)] antibodies were significantly associated with the presence of ABMR. Among ABMR cases, a higher level of anti-collagen type I [per 1 SD, adjusted hazard ratio (HR), 1.90 (1.32–2.75)] or type III per 1 SD, [adjusted HR, 1.57 (1.15–2.16)] antibody was associated with a higher risk of death-censored graft failure. In conclusion, post-transplant anti-collagen type I and type III antibodies may be novel non-HLA antibodies related to ABMR of kidney allografts.

## Graphical Abstract

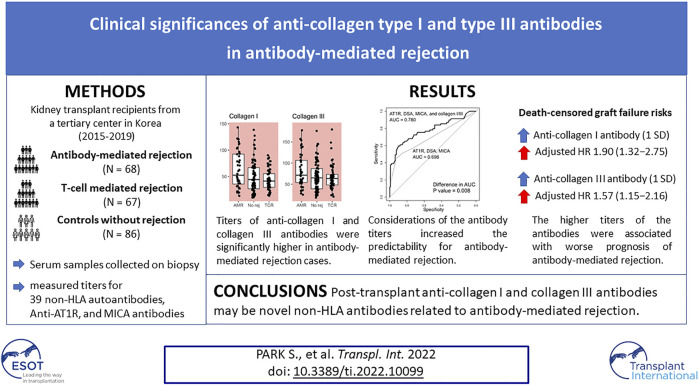

## Introduction

Kidney transplantation is the best treatment strategy for end-stage kidney disease. Although the overall prognosis of kidney transplantation has improved with the advances in potent immunosuppressive treatment strategies, the risk of graft loss in later periods after transplantation remains substantial. The majority of late graft failure cases are due to antibody-mediated rejection (ABMR), which has historically been described as chronic allograft nephropathy or transplant glomerulopathy (1, 2).

Currently, ABMR cases are diagnosed based on the presence of donor-specific antibodies (DSAs) against human leukocyte antigen (HLA) or non-HLA antigens and morphologic evidence of allograft injury represented by capillary injury, or glomerular inflammation (3). With the advances in anti-HLA antibody measurement methods, early detection and the management of preformed or *de-novo* DSAs has become possible. However, recent studies have revealed that a non-negligible portion of patients have histologic ABMR in the absence of HLA-DSAs (4).

In such HLA-DSA-negative histologic ABMR cases, the importance of non-HLA antibodies has been emphasized. Initially, anti-endothelial cell antibody was suggested to be formed during ischemia-reperfusion injury during organ transplantation, which accelerated ABMR even in the absence of HLA-DSA (5, 6). Later studies reported that angiotensin receptor I (AT1R) was a target antigen for non-HLA antibody in steroid-refractory vascular rejection cases with malignant hypertension (7). Autoantibodies against major histocompatibility complex class I chain-related antigens (MICA) have also been reported to have clinical significance for ABMR cases (8). Nevertheless, non-HLA antibodies remain understudied for their clinical significance in the kidney transplantation field (9). Further investigation is needed to identify novel non-HLA antibodies related to ABMR as there remain ABMR cases without detectable causal autoantibodies. Moreover, whether the presence of such non-HLA antibodies affects the prognosis of patients with ABMR needs to be assessed. Such evidence would enable clinicians to monitor and treat patients with ABMR in the early phases, which may reduce the risk of late graft failure in kidney transplant recipients.

In this study, we measured and compared the levels of 39 non-HLA antibodies in transplant recipients with ABMR, T-cell-mediated rejection (TCMR) cases, and control subjects without any evidence of rejection with the aim to identify a non-HLA antibody that can serve as a biomarker of ABMR and/or a predictor of prognosis. We hypothesized that by using an unsupervised approach, a novel non-HLA antibody with clinical significance in ABMR could be identified.

## Materials and Methods

### Ethical Considerations

This study was conducted in compliance with the Declaration of Helsinki and the Declaration of Istanbul. The institutional review board of Seoul National University Hospital, Seoul, Korea (H-1808-181-970) approved the study. All clinical characteristics and bio-specimens were prospectively collected with the approval of the study subjects.

### Study Cases

**FIGURE 1:**
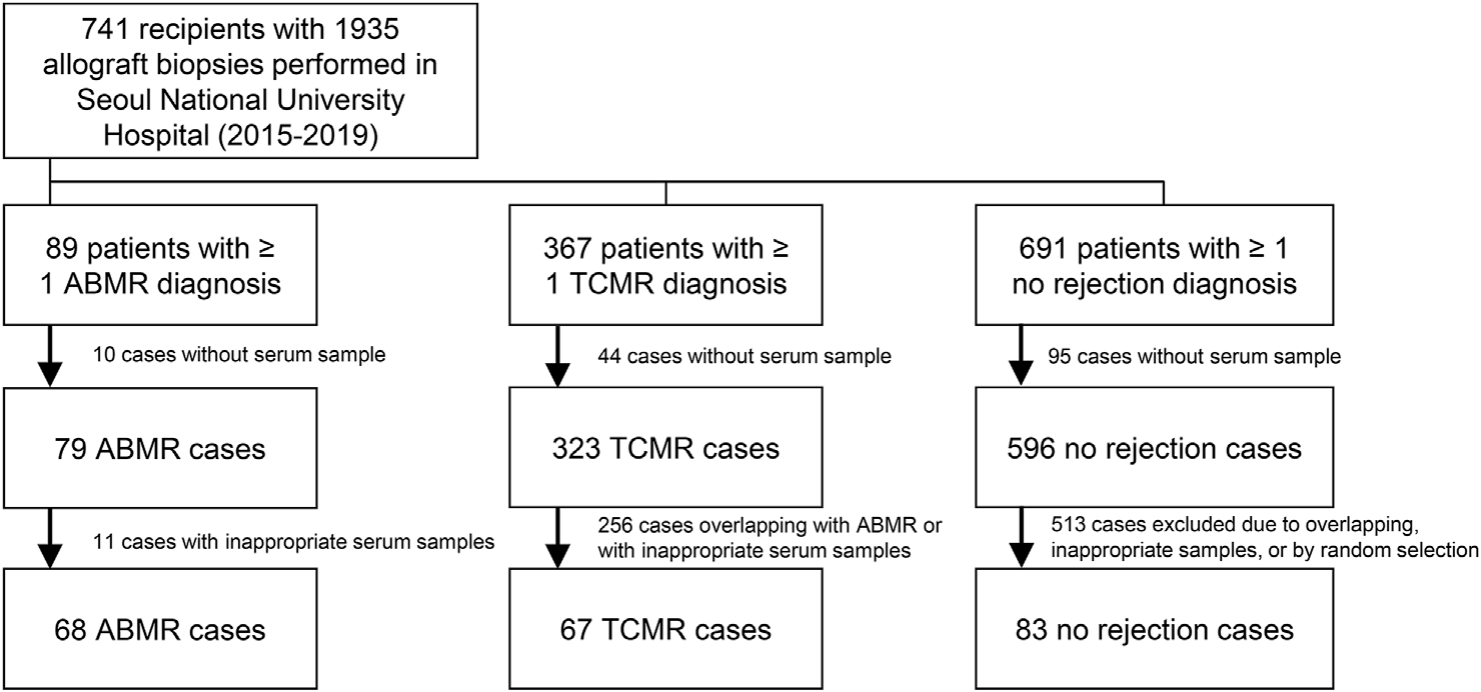
Study flow diagram.

The Seoul National University Hospital operates a human biobank for kidney transplant recipients and donors. In the biobank, serial samples (including serum, plasma, urine, and stool) collected (after acquiring informed consent from the patient) before transplantation, 2 weeks and 3 months after transplantation, and annually thereafter, if available, are stored. In addition, allograft biopsies were sampled from kidney transplant recipients. In the hospital, kidney biopsies were mostly performed based on the following clinical criteria: a progressive decline in renal function, persistent hematuria, or significant proteinuria of more than 1.0 g/day, and we collected pathologically confirmed ABMR cases as the main study group. There have been certain number of biopsy cases without any rejection, and such cases were mostly collected from protocol-based biopsies which were performed within short period from transplantation. In addition to ABMR and control cases without rejection, TCMR cases were collected as a rejection control group to identify non-HLA antibodies that are specifically associated with ABMR. We collected serum samples from non-overlapping 68 ABMR patients, 67 TCMR patients without ABMR, and 83 control subjects without any rejection confirmed by allograft biopsies between 2015 and 2019 at Seoul National University Hospital (Figure 1). ABMR or TCMR was distinguished based on the Banff classification, and 2013 and 2017 criteria were applied according to the time-periods (10–12). The samples were routinely reviewed by two kidney pathology specialists. Criteria for the selection of the ABMR cases were the availability of informed consent and of serum samples stored in the Seoul National University Hospital Biobank; no selection based on other clinical criteria was applied. The numbers of TCMR and control cases were determined to construct control groups of similar numbers based on random selection. We initially collected ABMR cases regardless of coexisting pathology (e.g., TCMR or calcineurin inhibitor toxicity) to maximize the number of cases.

### Antibody Screening Methods and Data Collection

We measured 39 non-HLA antibodies by the Luminex method using a commercial kit (LABScreen Autoantibody, One Lambda, CA, United States, URL: https://www.onelambda.com/en/product/labscreen-autoantibody-new.html, last accessed 2020-09-22) that reports mean fluorescence intensity (MFI) values. The targeted non-HLA antibodies in the kit were selected by the manufacturer based on a review of the literature in the transplantation field. The MFI values were calculated by subtracting the sample-specific fluorescence value for negative control beads from the sample-specific fluorescence value for non-HLA antigen beads. We additionally measured anti-MICA antibody levels (Luminex method, LABScreen Mixed, One Lambda) and anti-AT1R antibody levels (enzyme immunoassay, EIA-AT1RX, One Lambda) in the serum samples to determine the clinical significance of identified non-HLA antibodies independent from non-HLA antibodies that are known in the kidney transplantation field. The anti-MICA (normalized MFI ratio ≥2.7-fold) and anti-AT1R (≥10 U/mL) antibody test results were stratified as positive/negative according to the manufacturer’s recommended cutoff values. The other clinical data were collected through electronic medical record review, which are presented in Supplementary Methods. Serum samples and data of the ABMR or TCMR cases were collected at the timing of kidney biopsy which was performed to diagnosis the rejections.

### Internal Validation Study

We collected cases with available serum samples for internal validation by ELISA, including 26 ABMR cases and 28 controls (22 no rejection controls and 6 TCMR cases). The correlation between the anti-collagen I IgG antibody titers (unit/ml) measured by ELISA and the MFI values measured by the Luminex method was assessed by the Pearson’s correlation test, and the average or categorical values were compared between the two groups by t-test and the chi-squared test.

### Statistical Analysis

Non-HLA antibodies of which the levels significantly differed in the ABMR cases were selected as potential target biomarkers by the Mann-Whitney U test. We compared the characteristics between the subgroups and evaluated predictability by receiver-operating-characteristics (ROC) curves. The prognosis analysis was performed by Cox regression. Statistical significance was determined using a Bonferroni-corrected *p*-value (*p* < 0.05/39), and the 75th percentile values for ABMR, TCMR, and no-rejection cases were determined as cut-offs to determine a high non-HLA antibody titer. In other analyses, conventional two-sided *p*-values < 0.05 were considered significant. All statistical analyses were conducted using R (version 4.0.2, the R foundation). The other details of the statistical analysis are presented in Supplementary Methods.

## Results

### Study Population

The median duration from transplantation to biopsy was longer in the ABMR cases than in TCMR and no-rejection cases (Table 1). No-rejection cases were mostly from living, related donors. All cases had negative crossmatch results. Regarding lab findings, the ABMR group had the highest median serum creatinine values. The ABMR cases had a higher proportion of ABO-incompatible transplantation than the no-rejection group, but the proportion was smaller than that in the TCMR cases. Delayed graft function, which occurred in more than 10%, was more prevalent, and cold ischemic time was longer in the ABMR cases than in the other groups. Among the ABMR cases, biopsy specimens of 52 (76%) cases were positively stained for C4d, including 1+ (19 cases), 2+ (20 cases), and 3+ (13 cases) results, respectively. Among the TCMR cases, 48 cases did not have any v-lesions but t- and c-lesions (grade I TCMR). Of the remaining 19 cases with v-lesions, 12 TCMR cases were identified to have isolated v-lesions as they had minimal-to-no interstitial or microvascular inflammation and negative C4d staining results. There were 2 calcineurin inhibitor toxicity and 2 acute tubular necrosis cases diagnosed among the no rejection controls, otherwise, there were no pathologic diagnosis among the samples.

**TABLE 1:** Clinical characteristics of the study cases.

	ABMR group (N = 68)	No rejection control (N = 83)	TCMR group (N =67)
Age at diagnosis (years)	49.0 [39.5;58.5]	51.0 [39.5;58.0]	49.0 [34.5;55.0]
Sex			
Female	31 (45.6%)	35 (42.2%)	24 (35.8%)
Male	37 (54.4%)	48 (57.8%)	43 (64.2%)
Duration from transplantation to diagnosis (days)	73.5 [9.0;1002.5]	9.0 [9.0;11.0]	9.0 [9.0;11.5]
Duration from diagnosis to serum acquisition (days)	0.0 [ 0.0; 0.0]	0.0 [ 0.0; 0.0]	0.0 [ 0.0; 0.0]
Relation			
Living related	17 (25.0%)	42 (50.6%)	26 (38.8%)
Living unrelated	24 (35.3%)	20 (24.1%)	21 (31.3%)
Cadaveric	27 (39.7%)	21 (25.3%)	20 (29.9%)
Immunologic risk status			
ABO incompatibility	14 (21.2%)	19 (23.5%)	7 (10.4%)
HLA incompatibility at transplantation			
Crossmatch (+), DSA (+)	0 (0.0%)	0 (0.0%)	0 (0.0%)
Crossmatch (-), DSA (+)	9 (17.3%)	0 (0.0%)	0 (0.0%)
DSA on time of kidney biopsy	31 (45.6%)	5 (6.0%)	6 (9.0%)
Type			
Class I	11 (16.2%)	0 (0.0%)	0 (0.0%)
Class II	17 (25.5%)	0 (0.0%)	0 (0.0%)
Class I and class II	3 (4.4%)	5 (6.0%)	6 (9.0%)
Total MFI of those with DSA	7178 [736;21502]	2317 [961;2452]	1521 [1446;1584]
Peak MFI of those with DSA	6559 [736;20758]	1425 [961;2452]	1475 [878;1539]
Number of HLA mismatch	4 [3; 5]	3 [1; 4]	3 [2; 5]
Number of mismatches in HLA-A			
1	40 (65.6%)	41 (52.6%)	31 (47.7%)
2	12 (19.7%)	8 (10.3%)	13 (20.0%)
Number of mismatches in HLA-B			
1	19 (31.1%)	34 (43.6%)	26 (40.0%)
2	39 (63.9%)	24 (30.8%)	27 (41.5%)
Number of mismatches in HLA-DR			
1	27 (44.3%)	44 (56.5%)	31 (47.7%)
2	25 (41.0%)	9 (11.5%)	22 (33.8%)
Immunosuppressive treatment			
Desensitization	20 (29.4%)	22 (27.2%)	5 (7.5%)
Induction therapy			
Anti-thymocyte globulin	6 (25.0%)	8 (9.8%)	7 (10.4%)
IL-2 receptor subunit α inhibitor	50 (78.1%)	73 (88.0%)	67 (100.0%)
Maintenance immunosuppression			
Calcineurin inhibitors	65 (96.6%)	83 (100.0%)	65 (97.0%)
Tacrolimus	57 (83.8%)	83 (100.0%)	65 (97.0%)
Cyclosporine	8 (11.8%)	0 (0.0%)	0 (0.0%)
Mycophenolic acid	46 (67.6%)	77 (92.8%)	61 (91.0%)
Steroid	67 (98.5%)	83 (100.0%)	65 (97.0%)
Laboratory findings			
Serum albumin (g/dL)	3.7 [3.2; 4.0]	3.5 [3.3; 3.9]	3.6 [3.3; 3.8]
BUN (mg/dL)	31.0 [20.0;44.5]	19.0 [15.0;23.0]	22.5 [16.0;27.0]
Creatinine (mg/dL)	1.7 [1.1; 2.7]	1.0 [0.8; 1.4]	1.2 [0.9; 1.6]
Proteinuria (g/g or g/day)	0.8 [0.4; 1.7]	1.0 [0.4; 1.4]	1.0 [0.5; 1.6]
Blood pressures			
Systolic BP (mmHg)	135.0 [122.0;141.0]	128.0 [118.0;138.0]	127.5 [117.0;139.0]
Diastolic BP (mmHg)	85.0 [75.5;90.0]	82.0 [73.5;89.5]	82.0 [76.0;93.0]
Peri-transplant findings			
Delayed graft function	8 (13.1%)	3 (3.7%)	4 (6.0%)
Cold ischemic time (minutes)	54.0 [33.0;103.0]	3.0 [2.0; 3.0]	3.0 [2.0; 3.0]
Warm ischemic time (minutes)	41.0 [35.5;47.0]	79.0 [67.0;94.0]	73.5 [57.5;94.0]

Continuous values are presented as median [interquartile ranges] and categorical variables are presented as number (%). ABMR, antibody-mediated rejection; TCMR, T-cell mediated rejection; HLA, human leukocyte antigen; DSA, donor specific-antibody; PRA, panel reactive antibody; BP, blood pressure.

### Levels of Non-HLA Antibodies

Anti-AT1R antibodies were more frequently observed in the ABMR cases than in the TCMR or no-rejection control group. However, the proportion of patients testing positive for anti-MICA antibody was not significantly different among the three groups (Supplementary Table S1).

**FIGURE 2:**
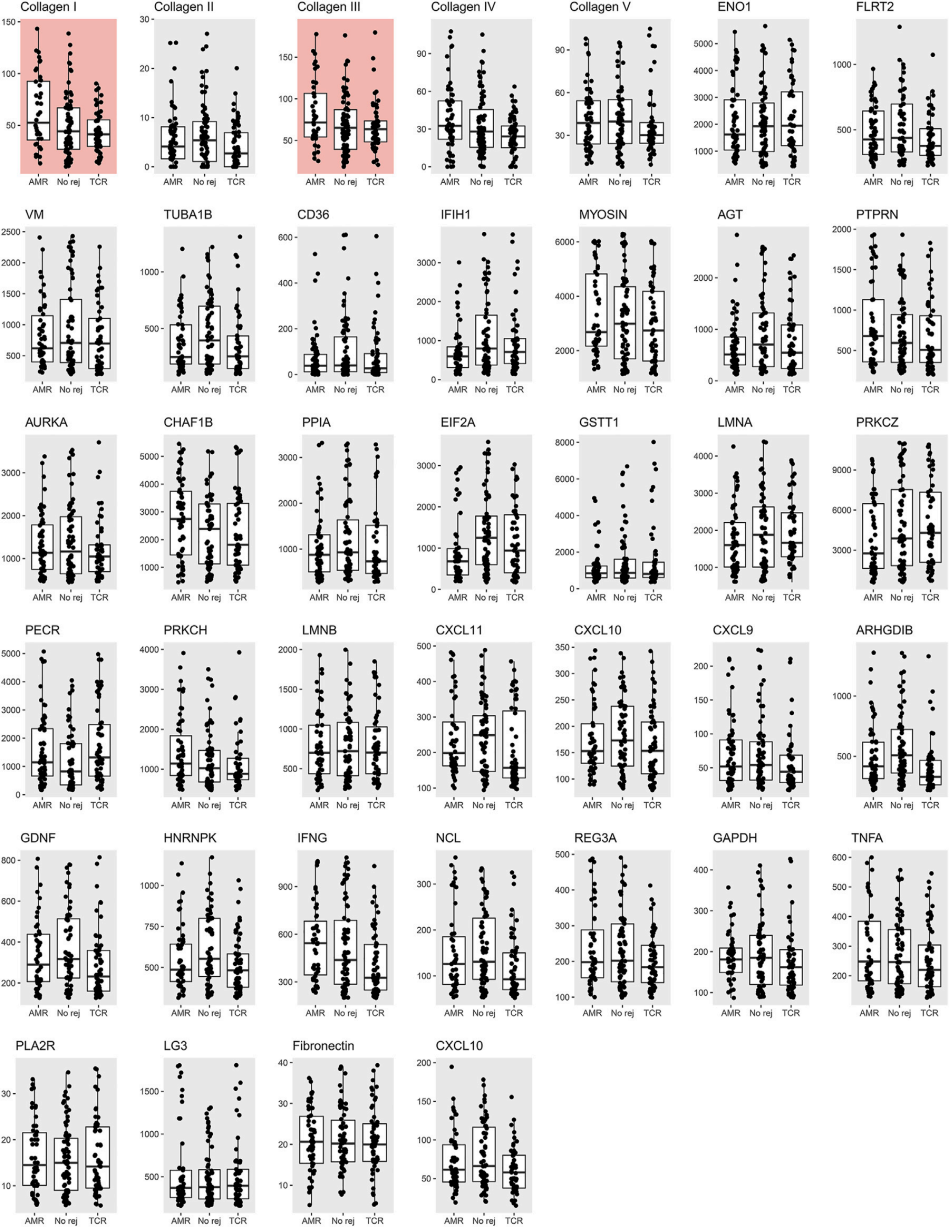
Measured non-HLA antibody levels among the antibody-mediated rejection, T-cell mediated rejection, and no rejection control groups. The median and interquartile values are presented by box and horizontal lines. The dots represent each level of a patient. The red background boxes for collagen I and III indicate that anti-collagen type I and type III antibody levels were significantly higher in the antibody-mediated rejection cases when compared to the T-cell mediated rejection and no rejection controls.

**FIGURE 3:**
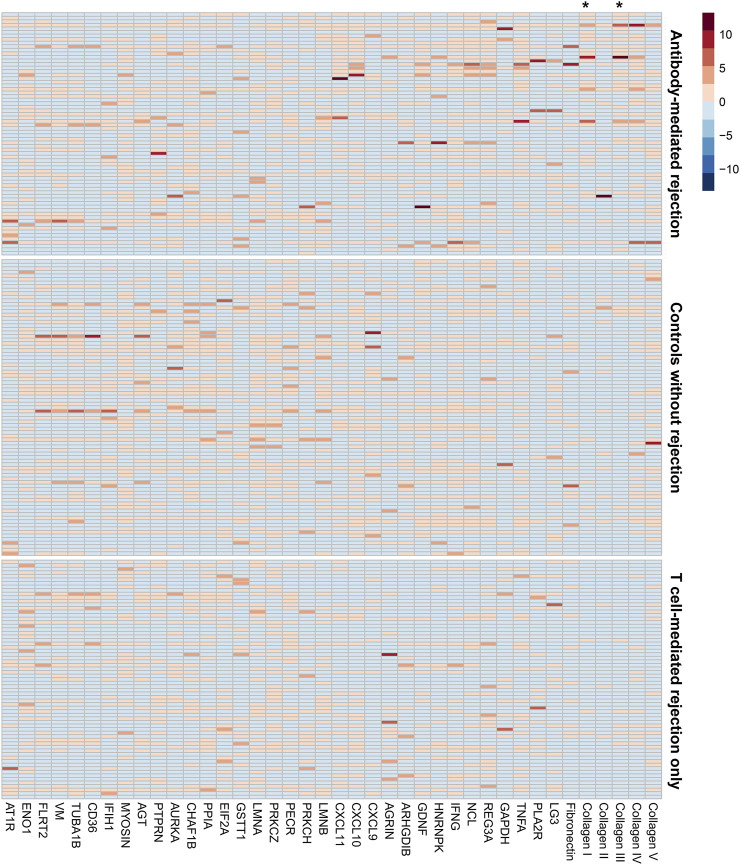
The heatmap presenting the individual-level relative levels of each non-HLA antibody. The names of the antigens are shown in column headings. Each row is the result from an individual in the study groups. The three groups, antibody-mediated rejection, no rejection control, and T cell rejection only, are marked in the right side of the figure. Asterisks (*) mark the column showing the results for anti-collagen type I and type III antibodies. The distribution of the non-HLA antibody levels were stratified into 0th (light blue) to 10th (dark red) deciles.

The antibody screening results are shown in Figure 2, which shows the comparison between the study groups, and Figure 3, which shows a heatmap presenting the relative levels in each sample, and Supplementary Table S2, which shows the statistical test results for differences in median values. We found that anti-collagen type I and anti-collagen type III antibodies were significantly higher in the ABMR cases than in the TCMR and no-rejection cases (*p* < 0.05/39). Differences in other antibodies did not reach the significance level when compared with the control groups. Moreover, when we stratified the antibody levels according to upper quartile (≥75th percentile) cut-offs among the ABMR, TCMR, and no-rejection cases, the proportions of recipients who had upper quartile ranges for anti-collagen type I and III antibodies were significantly higher among the ABMR patients than in the controls (Supplementary Table S3). Thus, anti-collagen type I and type III antibodies were selected for further analysis as target antibodies that may have clinical significance for ABMR. The antibody levels of anti-collagen type I and type III were strongly correlated (Supplementary Figure S1).

### Factors Associated With the Target Antibody Levels

Within the ABMR cases, those with high anti-collagen type I or type III levels had higher proportion of cadaveric transplantation cases and, thus, lower proportion of ABO incompatible cases (Supplementary Tables S4, S5). Otherwise, the transplantation characteristics were generally similar according to presence of high anti-collagen type I or type III levels, except for that those with high anti-collagen type III titers were more sensitized according to the results from PRA class II screenings.

We found that anti-collagen type I and type III antibody levels did not differ depending on the presence of HLA-DSAs, chronic-active lesions or coexisting TCMR or calcineurin inhibitor toxicity (Supplementary Tables S6–S8). The cold ischemic time showed a significant association with the anti-collagen type I antibody level, but was marginally associated with the anti-collagen type III antibody level (Supplementary Table S9). As for the relation with pathologic parameters (Supplementary Table S10), we found patients with a higher anti-collagen type I or III level more commonly had higher scores for peritubular capillaritis (ptc). Further, patients with a higher anti-collagen type I level more commonly had positive findings for interstitial inflammation (i). When we investigated the correlation between anti-collagen type I or type III antibody titer and the Banff lesions, again, ptc was identified to be significantly correlated with the titers (Supplementary Figure S3). Finally, when we compared anti-collagen type I and type III antibody levels of the 47 ABMR cases with the levels before transplantation measured in those with available samples, we found no significant differences in the median values or proportion of patients with high levels for anti-collagen type I and type III antibodies (Supplementary Table S11).

### Predictability of Antibody-Mediated Rejection

**FIGURE 4:**
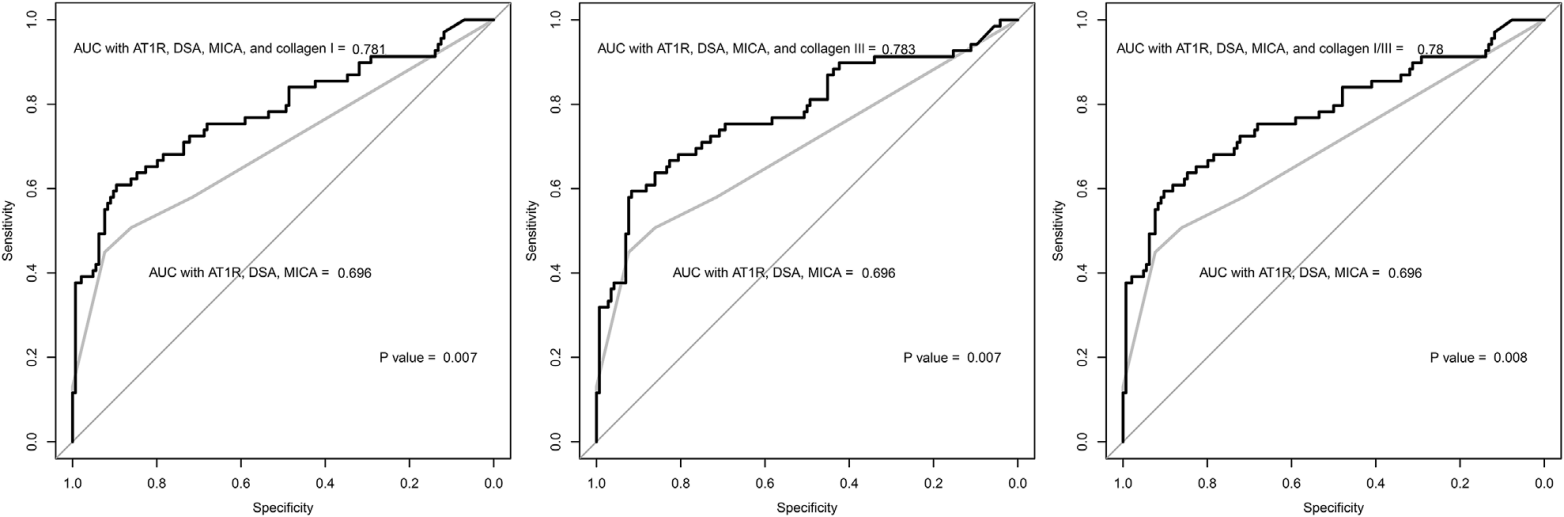
The receiver-operating-characteristics curve of the logistic regression models constructed with clinical variables associated with antibody-mediated rejection. The grey lines indicate the regression model including the following variables: presence of anti-AT1R, anti-MICA, and HLA-DSA antibodies. The black lines indicate the regression models including the levels of anti-collagen type I or type III antibody. The P values were calculated by the De Long’s method, and the results showed that the regression models including the anti-collagen type I or type III antibody level showed higher discriminative power than the model without the levels. AUC = area under curve.

The anti-collagen type I and type III antibody levels showed a positive association with the probability of ABMR occurrence in the studied patients (Supplementary Figure S2). A one standard deviation increase in the levels of both antibodies was associated with approximately 10-fold higher odds for ABMR in the univariable analysis (Table 2). In the multivariable analysis, anti-collagen type I or type III antibody levels and odds for ABMR were again significantly correlated. Female sex, presence of HLA-DSAs, longer duration from transplantation to biopsy, and higher serum creatinine values were other significant factors associated with a higher probability of ABMR occurrence. Further, addition of the anti-collagen type I antibody level to the ROC model including presence of HLA-DSA, anti-AT1R antibody, and anti-MICA antibody significantly improved the AUC values (0.781 vs. 0.696, *p* = 0.007) (Figure 4). Similarly, addition of the anti-collagen type III antibody level (0.783 vs. 0.696, *p* = 0.007) or of both anti-collagen type I and III antibody levels (0.780 vs. 0.696, *p* = 0.008) significantly improved the AUC values.

**TABLE 2:** Regression analysis to investigate the association between HLA or non-HLA antibody levels and odds for ABMR.

Exposure	Univariable model		Multivariable model^a^	
	OR for ABMR (95% CI)	P	Adjusted OR for ABMR (95% CI)	P
Anti-collagen type I antibody^b^				
continuous (1 SD increase)	12.92 (3.82–59.58)	<0.001	11.72 (2.73–76.30)	0.003
categorical (≥75 percentile)	5.64 (2.94–11.08)	<0.001	5.43 (2.24–13.70)	<0.001
Anti-collagen type III antibody^b^				
continuous (1 SD increase)	9.58 (3.33–36.11)	<0.001	6.22 (1.91–31.75)	0.01
categorical (≥75 percentile)	5.33 (2.80–10.39)	<0.001	1.53 (0.71–3.56)	0.28
HLA-DSA (yes vs. no)	9.28 (4.41–20.85)	<0.001	6.88 (2.69–18.60)	<0.001
Anti-AT1R antibody (yes vs. no)	3.58 (1.53–8.69)	0.004	2.46 (0.57–10.07)	0.21
Anti-MICA antibody (yes vs. no)	1.00 (0.44–2.15)	0.996	0.74 (0.18–2.51)	0.65

OR, odds ratio; ABMR, antibody-mediated rejection; CI, confidence interval; SD, standard deviation.

The effect sizes for HLA-DSA, anti-AT1R antibody, and anti-MICA antibody were from the model which was adjusted with the anti-collagen type I antibody levels (continuous).

The effect sizes of the other variables that reached significance level in the multivariable model was male sex [adjusted OR 0.31 (0.12–0.75), *p* = 0.01], serum creatinine [1 mg/dl increase, adjusted OR 2.56 (1.75–4.00), *p* < 0.001], and duration from transplantation to biopsy [30 days increase, adjusted OR 1.02 (1.002–1.03), *p* = 0.045]. The variables reaching statistically significant level was the same when the anti-collagen type III antibody level was included in the multivariable model.

a Multivariable model included age, sex, presence of HLA-DSA, anti-AT1R antibody, and anti-MICA, antibody at the time of allograft biopsy, duration from transplantation to allograft biopsy, donor types (living or deceased), serum creatinine levels, and anti-collagen type I or type III, antibody (each).

b The analysis was separately performed including continuous levels and categorically defined high levels as the exposures.

### Prognosis Analysis

**FIGURE 5:**
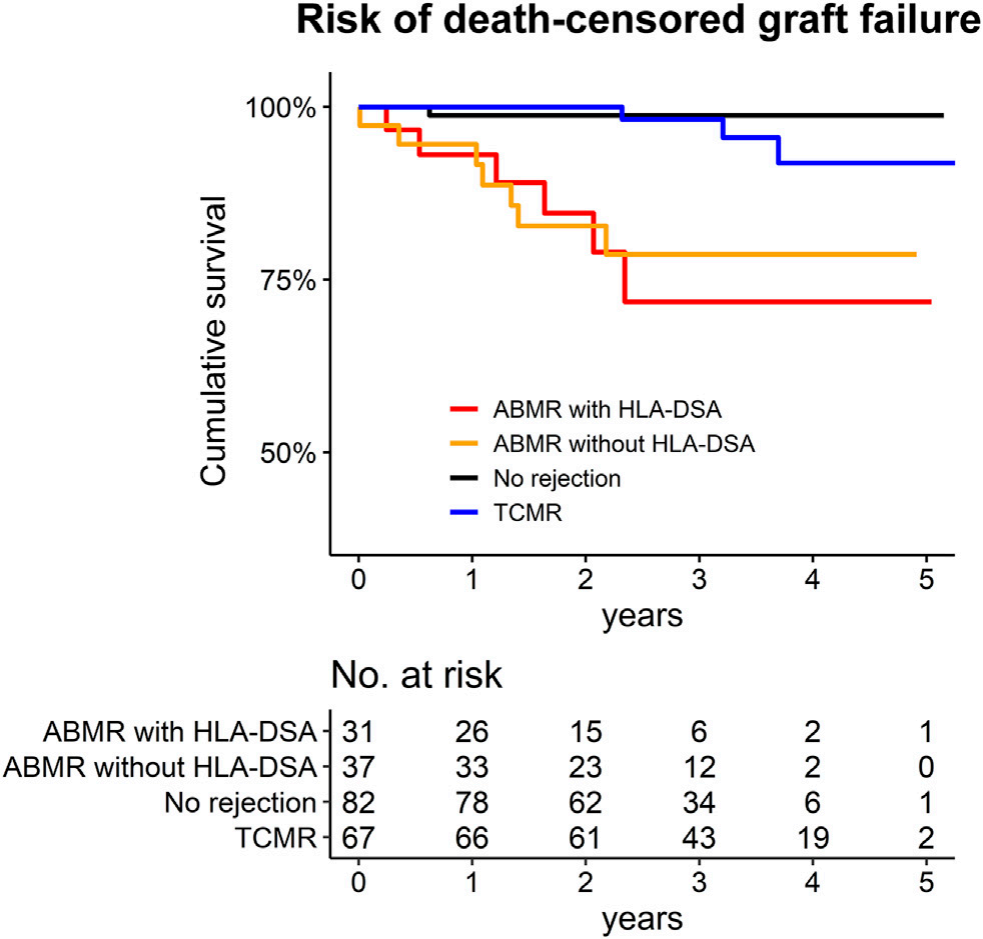
Kaplan Meier survival curve for the death-censored graft failure of the study population. The number at risk are presented below the graph.

In the ABMR group, 13 patients progressed to death-censored graft failure during a median of 2.1 [1.3–3.1] years of follow-up (Figure 5). There were two cases of death-with-graft function for which the follow-up was censored with the event. In the no-rejection controls, only one patient progressed to death-censored graft failure. In the TCMR group, there were three events of death-censored graft failure. Both patients with ABMR with HLA-DSA and those with ABMR without HLA-DSA had a significantly worse prognosis than the no-rejection controls (Supplementary Table S12). This significant difference remained when we used the TCMR cases as the reference group; ABMR cases, regardless of the presence of HLA-DSA, showed a significantly higher (>4-fold) hazard for death-censored graft failure than the TCMR patients.

When we evaluated the prognostic significance of the target antibodies among the ABMR cases, a one standard deviation increase in the anti-collagen type I or type III antibody level was associated with a significantly higher risk of death-censored graft failure (Table 3). The significance remained after multivariable adjustment for age, serum creatinine, presence of a mixed TCMR, and presence of HLA-DSA at the time of ABMR diagnosis, and a one standard deviation increase in the antibody level was associated with more than 50% higher risk of death-censored graft failure. In subgroups stratified by a high anti-AT1R antibody level (≥10 U/mL) or anti-collagen antibody levels, both antibodies showed potential prognostic significance, with higher levels being associated with a higher hazard of death-censored graft failure. Although statistical significance was not observed in the univariable model, after adjusting the baseline variables, patients with high levels of both anti-AT1R and anti-collagen type I and type III antibody showed a significantly higher risk of death-censored graft failure.

**TABLE 3:** Risk of death-censored graft failure of the ABMR group according to the levels of anti-collagen type I or III antibody.

Exposure	Univariable model		Multivariable model^a^	
	HR (95% CI)	P	Adjusted HR (95% CI)	P
Anti-collagen type I antibody level				
1 standard deviation increase	1.65 (1.24–2.19)	<0.001	1.90 (1.32–2.75)	<0.001
>1st tertile value within ABMR cases	5.00 (1.89–13.25)	0.001	5.99 (1.16–30.91)	0.03
Subgroup divided by anti-AT1R antibody and anti-collagen type I antibody level ^b^				
Low anti-AT1R antibody level and low anti-collagen type I antibody level	Reference		Reference	
Low anti-AT1R antibody level and high anti-collagen type I antibody level	1.47 (0.33–6.57)	0.62	1.77 (0.32–9.38)	0.51
High anti-AT1R antibody level and low anti-collagen type I antibody level	4.21 (0.85–20.86)	0.08	4.44 (0.86–22.75)	0.07
High anti-AT1R antibody level and high anti-collagen type I antibody level	4.18 (0.84–20.74)	0.08	8.48 (1.08–66.40)	0.04
Anti-collagen type III antibody level				
1 standard deviation increase	1.44 (1.10–1.88)	0.007	1.57 (1.15–2.16)	0.005
>1st tertile value within ABMR cases	1.23 (0.41–3.65)	0.713	1.76 (0.44–6.99)	0.42
Subgroup divided by anti-AT1R antibody and anti-collagen type III antibody level^b^				
Low anti-AT1R antibody level and low anti-collagen type III antibody level	Reference		Reference	
Low anti-AT1R antibody level and high anti-collagen type III antibody level	1.11 (0.25–4.98)	0.89	1.44 (0.28–7.52)	0.66
High anti-AT1R antibody level and low anti-collagen type III antibody level	3.60 (0.60–21.56)	0.16	3.74 (0.59–23.69)	0.16
High anti-AT1R antibody level and high anti-collagen type III antibody level	3.65 (0.82–16.37)	0.09	6.70 (1.05–43.03)	0.04

HR, hazard ratio; CI, confidence interval; SD, standard deviation.

a Multivariable model was adjusted for age and serum creatinine values, presence of a mixed T-cell mediated rejection, and presence of any HLA-DSA at the time of ABMR diagnosis.

b High level of anti-AT1R, antibody was determined as ≥ 10 U/mL. Anti-collagen type I or type III antibody level was recategorized as > 1st tertile value. The alternate threshold for anti-collagen type I or type III antibody was applied because a Cox regression model was not constructed for certain analysis when 75 percentile value was applied because there was a subgroup with zero outcome.

### Internal Validation

The OD-based anti-collagen I IgG antibody values measured by ELISA and the MFI titers measured by the Luminex method showed significant (*p* < 0.001) correlation with each other (Pearson R = 0.580, Supplementary Figure S4). In addition, the ABMR group showed average 44.9 ± 57.5 unit/ml of anti-collagen I IgG antibody measured by ELISA, which was significantly (*p* = 0.040) higher than that of the controls (20.4 ± 21.6 unit/ml). Those with high (>75 percentile) anti-collagen I antibody titers measured by the Luminex method were significantly associated with higher values (>75 percentile) measured by ELISA (8/15, 53.3%), while those with the lower ranges of titers by the Luminex method also frequently showed low values by ELISA (33/39, 84.6%) (*p* = 0.003).

## Discussion

In this study, we measured serum levels of 39 non-HLA antibodies in patients with biopsy-confirmed ABMR, TCMR, and absence of rejection, which revealed that anti-collagen type I and type III antibody levels were significantly elevated particularly in the ABMR patients. The addition of the anti-collagen type I or III antibody level significantly improved the predictability of models for ABMR including the presence of HLA-DSA or other previously reported non-HLA antibodies. Further, patients with a high anti-collagen type I or III antibody level showed a worse prognosis. Thus, anti-collagen type I or III antibody may be a biomarker with diagnostic and prognostic value for ABMR.

ABMR currently is a major cause of late graft failure in the transplantation field. Although the identification of the significance of HLA-DSA has enabled the development of therapeutic strategies for the prevention, monitoring, and treatment of a portion of ABMR patients, a non-negligible portion of ABMR patients is HLA-DSA-negative (1, 4) Further, previous studies on ABMR pathology or using allograft transcriptome profiling could not clearly distinguish HLA-DSA-negative ABMR from HLA-DSA-positive cases (4, 13), thus, additional serologic biomarkers may be helpful to diagnose ABMR. Considering the urgent need for biomarkers to aid the diagnosis of ABMR, the recent 2017 Banff classification included C4d deposition, intrarenal transcriptomic findings associated with ABMR, and circulating non-HLA antibodies as surrogate markers for ABMR (10). Nevertheless, non-HLA antibodies show wide ranges, and specific non-HLA antibodies associated with ABMR needs to be further clarified (9). One strength of our study was that we determined the levels of various non-HLA antibodies in a relatively large number of ABMR cases and compared them with those in both pure TCMR and no-rejection control cases. The aim of this approach was to identify a non-HLA antibody that is associated with the presence of ABMR in allograft biopsy. Indeed, we successfully identified anti-collagen type I and type III antibodies as being related to ABMR independent of the presence of HLA-DSA, anti-AT1R, or anti-MICA antibody. In addition, the HLA-DSA-negative ABMR patients showed significantly worse prognosis than TCMR patients or no-rejection controls, which was different from findings in previous reports (4, 13). This further highlights the necessity of additional biomarkers for ABMR. Even the prognosis of ABMR was different according to the antibody levels; a higher level of anti-collagen type I or type III antibody was associated with a higher risk of death-censored graft failure. Thus, our findings suggest the potential diagnostic and prognostic value of anti-collagen type I and type III antibody levels for kidney transplant recipients with a suspected risk of rejection.

A high collagen turnover has been suggested as a marker for certain kidney pathologies (14). Enzyme-degraded collagen molecules have been associated with ischemia-reperfusion injury, which has been suggested to be the cause of anti-endothelial cell antibody production in ABMR (15). Clinically, high collagen turnover, detected from urine, has been reported in immunoglobulin A nephropathy (14), interstitial fibrosis of kidney transplant recipients (16), or kidney fibrosis in chronic kidney disease (17, 18). In lung transplantation, collagen type V present in airway epithelial cells is the antigen of non-HLA antibody associated with pulmonary graft injury (19). Collagen type V is important for cardiovascular organs, and anti-collagen type V has been reported to be related to ABMR in heart transplantation (20). Further, the fact that collagen type I and type III molecules are the abundant collagen types in kidneys supports the relevance of our findings regarding ABMR in kidney allografts (21). We observed that anti-collagen type I or type III antibody titer was particularly associated with peritubular capillaritis (ptc) and the collagen molecules are present in kidney microvascular structure. Thus, anti-collagen type I antibody may be a measurable marker of extracellular matrix remodeling or endothelial damage, which occurs in ABMR (22). As anti-collagen type III level was not associated with such relevant findings, anti-collagen type I antibody may be prioritized for further investigation for the significance in ABMR.

Our study could not confirm whether the high anti-collagen type I and type III antibody levels have a causal effect to ABMR. The finding that the anti-collagen type I antibody level was higher in ABMR cases than in the donor controls and was associated with longer cold ischemic time may support that the formation of the antibodies during transplant surgery might have caused ABMR. Considering that collagen I or III molecule would be present in kidney microvascular structure, the exposure of neoepitope during transplant surgery by ischemic-reperfusion injury may cause formation of anti-collagen autoantibodies, further contributing to the development of ABMR. Or, preformed anti-collagen type I and type III antibodies may bind to the transplanted graft and cause ABMR. However, the cold ischemic time information was available in the limited portion of patients and the timing of serum collection was heterogeneous, so confirmation of the hypothesis was hardly possible. Among the available samples, pre-transplant levels of anti-collagen type I or type III were not different from post-transplant levels, thus, it is possible that these antibodies may simply be a surrogate biomarker reflecting the fibrotic change in the ABMR pathology. Additional study is warranted to investigate whether these antibodies may cause ABMR and the mechanistical background.

Our study has several limitations. First, whether the levels of anti-collagen type I and type III antibodies change after treatment strategies for ABMR (e.g., plasmapheresis or high-dose immunosuppression) along with improvement in allograft function or a serial sample investigation for kinetics of autoantibody development was not studied. Such information would support that anti-collagen type I or type III antibody may be a novel causal non-HLA antibody that facilitates ABMR. Second, further experimental validation is necessary to determine the direct effects of anti-collagen type I and type III antibodies on the allograft. Third, the study was performed in a single center, implying a possibility of selection bias, although we randomly selected cases with available serum specimens. Additional validation in an independent cohort is warranted to confirm the clinical significance of anti-collagen type I and type III antibodies. Fourth, because of the selection bias and a modest sample size, clinical significance of other non-HLA antibodies might not have been observed due to false negative bias. Therefore, the null findings of our study may not preclude the possibility that other non-HLA antibodies may be related to development of ABMR. Lastly, the study patients were of Asian ethnicities, which have distinct peri-transplant characteristics from other ethnicities; thus, our study findings cannot be generalized.

In conclusion, among measured 39 non-HLA antibodies, anti-collagen type I and type III antibody levels were significantly higher in ABMR cases. Higher levels of these two antibodies were associated with a higher risk of death-censored-graft failure in ABMR. The mechanisms of action of anti-collagen type I and type III antibodies on kidney allograft need to be investigated in future studies.

## Data Availability

The raw data supporting the conclusion of this article will be made available by the authors, without undue reservation.

## Abbreviations

ABMR, antibody-mediated rejection; DSA, donor specific antibody; HLA, human leukocyte antigen; TCMR, T-cell mediated rejection.
