# Supplementary material for: Clinical Significances of Anti-Collagen Type I and Type III Antibodies in Antibody-Mediated Rejection
Source: Transpl Int. 2022 May 11;35:10099. doi: 10.3389/ti.2022.10099 (PMC9131656; doi:10.3389/ti.2022.10099)
Supplement: Supplementary file 1 [file DataSheet1.pdf]

Clinical significances of anti-collagen type I and type III antibodies in antibody-mediated rejection

Sehoon Park, Seung-Hee Yang, Jiyeon Kim, Semin Cho, Jaeseok Yang, Sang-il Min, Jongwon Ha, Chang Wook Jeong, Seong Hee Bhoo, Yong Chul Kim, Dong Ki Kim, Kook-Hwan Oh, Kwon Wook Joo, Yon Su Kim, Kyung Chul Moon, Eun Young Seong, Hajeong Lee

Supplemental Material Table of Contents

Supplemental Methods.

Supplemental Table 1. Previously reported non-HLA antibodies in the study cases.

Supplemental Table 2. Screening for non-HLA antibodies that were elevated in ABMR cases

Supplemental Table 3. The proportion of kidney transplant recipients with the upper quartile ranges (≥ 75 percentile) of according to antibody levels.

Supplemental Table 4. Clinical characteristics of the ABMR cases according to presence of high (≥ 75 percentile) anti-collagen type I level.

Supplemental Table 5. Clinical characteristics of the ABMR cases according to presence of high (≥ 75 percentile) anti-collagen type III level.

Supplemental Table 6. Non-HLA antibody levels according to presence of HLA-DSAs.

Supplemental Table 7. Non-HLA antibody levels according to presence of chronic-active AMR.

Supplemental Table 8. Non-HLA antibody levels according to coexistence of TCMR or calcineurin inhibitor toxicity.

Supplemental Table 9. Association between ischemic time and anti-collagen I and III level (MFI) within the antibody mediated rejection group.

Supplemental Table 10. Banff classification according to levels of anti-collagen type I or type III antibodies.

Supplemental Table 11. Comparison of the anti-collagen type I and type III antibody levels before and after transplantation in 47 ABMR cases.

Supplemental Table 12. Risk of death-censored graft failure of the subgroups.

Supplemental Figure 1. Correlation between anti-collagen type I and type III antibody level.

Supplemental Figure 2. Investigation for the the association between non-HLA antibody level and odds for ABMR.

Supplemental Figure 3. The correlation between anti-collagen type I or type III antibody titers and Banff lesions, including both ABMR and TCMR cases.

Supplemental Figure 4. The correlation between the antibody values measured by ELISA method and the MFI titers measured by the bead-based Luminex method.

Supplemental Methods

Clinical Data collection

We collected the following data from the transplant recipients: age, sex, duration from transplantation to kidney biopsy, duration from biopsy to serum acquisition, relation to donors, sensitization-related status (i.e., ABO incompatibility and the presence of preformed HLA-DSAs determined in the pre-transplant period), usage of maintenance immunosuppressive agents, induction immunotherapy implemented at the time of transplantation, whether the transplantation was a desensitized case, and laboratory findings at the timing of biopsy, including blood pressure, serum protein, albumin, blood urea nitrogen (BUN), serum creatinine, and proteinuria amount (by spot urine protein-to-creatinine ratio or 24-h-collected urine samples), and whether there was a delayed graft function event. In patients with surgery-related records, available information of cold and warm ischemic times was collected. We collected the death-censored graft failure outcomes of the ABMR cases to determine the prognosis.

Details regarding the statistical analysis

We first compared the levels of non-HLA antibodies between the study groups. Non-HLA antibodies of which the levels significantly differed in the ABMR cases were selected as potential target biomarkers by the Mann-Whitney U test. Statistical significance was determined using a Bonferroni-corrected P-value ($P < 0.05/39$), and the 75th percentile values for ABMR, TCMR, and no-rejection cases were determined as cut-offs to determine a high non-HLA antibody level.

Next, as some ABMR cases had coexisting pathological findings, mostly from TCMR or calcineurin inhibitor toxicity, we evaluated whether target non-HLA antibody levels within the ABMR group differed according to the coexistence of TCMR or calcineurin inhibitor toxicity. Further, we evaluated whether target non-HLA antibody levels within the ABMR group differed according to the presence of HLA-DSAs, and whether the Banff classification scores differed according to the target non-HLA antibody level. For patients with available information on the ischemic time, whether the cold or warm ischemic time was significantly associated with the target non-HLA antibody level was studied by linear regression analysis. Univariable and multivariable models were constructed, and the multivariable model was adjusted for age, serum creatinine, the presence of non-HLA-DSAs, and duration from transplantation to biopsy given the clinical importance of these variables. Additionally, target antibody levels were compared with those in the donors. Antibody levels before and after transplantation were compared using a paired t-test.

Further, whether the odds of ABMR showed a positive association with target non-HLA antibody levels was investigated using a generalized additive model. Additionally, logistic regression analyses were conducted. Univariable and multivariable models were constructed, and the multivariable model was adjusted for age, serum creatinine, presence of non-HLA-DSAs, duration from transplantation to biopsy, donor relation (living or deceased), presence of positive anti-AT1R, and anti-MICA antibodies to test whether the clinical significance of target non-HLA antibody was present independent from age, kidney function, duration from transplantation, donor types, and previously reported antibody biomarkers for ABMR. In addition, receiver-

operating-characteristics (ROC) curve analysis was performed. Whether the addition of the target non-HLA antibodies to the prediction model for ABMR among all study patients improved the discriminatory power of the conventional model, including the presence of HLA-DSA and anti-AT1R or anti-MICA antibodies, was investigated by comparing area-under-curve (AUC) values by the DeLong method.

For prognostic analysis, the death-censored graft failure prognosis was investigated based on Kaplan-Meier survival curves. The association between the target non-HLA antibody levels and the risk of death-censored raft failure was investigated by Cox regression analysis. Univariable and multivariable models were constructed. The adjusted variables in the multivariable models included age, serum creatinine, and the presence of non-HLA-DSAs at the time of biopsy. The categorical antibody level was recategorized to > first tertile level. The number of adjusted variables was limited as the outcomes occurred in only few cases.

Supplemental Table 1. Previously reported non-HLA antibodies in the study cases.

Exposure	ABMR group (N = 68)	No rejection controls (N = 83)	TCMR group (N =67)	P
Anti-AT1R antibody				0.004
negative	53 (77.9%)	78 (94.0%)	62 (92.5%)	
positive	15 (22.1%)	5 (6.0%)	5 (7.5%)	
Anti-MICA antibody				0.71
negative	54 (83.1%)	71 (86.6%)	54 (81.8%)	
positive	11 (16.9%)	11 (13.4%)	12 (18.2%)	

ABMR = antibody-mediated rejection, TCMR = T-cell mediated rejection

There were missing in anti-MICA antibody levels in 3 ABMR cases, 1 no rejection control case, and 1 TCMR case.

Supplemental Table 2. Screening for non-HLA antibodies that were elevated in ABMR cases

Antigen for non-HLA antibody	ABMR group (N = 68)	No rejection controls (N = 83)	TCMR group (N = 67)	P (ABMR vs. no rejection)	P (ABMR vs. TCMR)
Collagen I	74.2 [30.2;183.0]	43.4 [23.6;66.6]	38.9 [24.8;56.8]	0.001	0.001
Collagen II	5.3 [1.7;12.1]	6.1 [2.0;11.9]	3.0 [0.0; 7.5]	0.912	0.016
Collagen III	93.4 [53.5;216.2]	58.9 [35.5;81.1]	59.6 [43.1;73.2]	< 0.001	< 0.001
Collagen IV	34.3 [23.4;63.2]	28.6 [15.8;48.5]	24.5 [15.6;33.4]	0.016	< 0.001
Collagen V	41.5 [22.9;61.3]	40.5 [19.6;61.6]	28.7 [21.2;39.1]	0.663	0.027
AGRIN	54.8 [30.5;103.0]	50.7 [26.1;95.6]	48.0 [26.4;91.6]	0.498	0.357
AGT	512.8 [290.1;951.4]	642.2 [204.0;1674.8]	546.2 [225.0;1198.8]	0.347	0.9
ARHGDIB	421.5 [309.4;688.3]	512.0 [338.3;882.7]	316.8 [243.4;476.4]	0.252	0.013
AURKA	1155.4 [715.0;1959.5]	1281.3 [622.5;2228.8]	1014.8 [536.6;1421.7]	0.957	0.088
CD36	41.7 [14.5;113.8]	47.6 [14.7;234.0]	30.9 [9.8;151.3]	0.175	0.848
CHAF1B	2680.4 [1106.6;3879.0]	2526.0 [984.5;3822.7]	1811.9 [955.9;3432.6]	0.999	0.296
CXCL10	160.6 [120.2;274.5]	175.6 [107.0;257.5]	131.6 [104.0;207.2]	0.897	0.045
CXCL11	232.0 [160.5;468.0]	225.4 [135.9;315.2]	154.1 [124.3;324.3]	0.092	0.001
CXCL9	61.5 [42.3;103.8]	71.0 [44.8;127.3]	50.2 [32.2;81.9]	0.262	0.074
EIF2A	694.0 [302.5;2015.9]	1185.0 [481.2;2210.9]	941.7 [322.6;1897.9]	0.1	0.531
ENO1	1595.2 [894.1;3272.6]	1823.1 [844.2;3058.5]	1996.2 [1113.7;4162.1]	0.639	0.315
Fibronectin	21.4 [15.3;30.5]	20.3 [14.3;29.3]	19.3 [13.2;24.8]	0.462	0.048
FLRT2	456.6 [309.0;694.9]	437.8 [267.3;802.6]	371.5 [268.5;584.0]	0.996	0.077
GAPDH	186.8 [148.3;249.6]	183.0 [104.2;243.6]	161.6 [107.3;219.6]	0.29	0.041
GDNF	323.2 [193.0;557.1]	321.0 [176.3;548.7]	209.5 [148.5;358.3]	0.894	0.011
GSTT1	863.8 [582.2;1373.8]	815.6 [446.0;1930.4]	847.6 [590.4;2845.5]	0.593	0.828
HNRNPK	499.5 [392.0;809.1]	536.1 [367.6;825.7]	482.2 [372.6;606.2]	0.979	0.18
IFIH1	597.0 [223.5;1596.7]	815.0 [322.4;2257.0]	677.2 [315.7;1148.1]	0.153	0.655
IFNG	546.8 [289.4;842.5]	454.0 [273.7;827.1]	319.2 [231.4;541.5]	0.497	0.006
LG3	371.2 [241.6;1035.9]	380.2 [221.5;776.5]	392.3 [206.9;676.7]	0.809	0.674
LMNA	1604.8 [968.5;2505.0]	1922.9 [880.3;3149.2]	1653.0 [1169.8;2621.6]	0.512	0.488
LMNB	716.9 [420.0;1296.6]	660.0 [352.6;1160.8]	690.1 [357.7;1117.8]	0.397	0.385
MYOSIN	2683.4 [1909.9;5412.5]	3026.0 [1669.6;4557.3]	2605.7 [1367.6;4355.4]	0.743	0.145
NCL	134.0 [78.5;319.2]	126.5 [78.9;230.7]	84.7 [63.2;152.1]	0.416	0.005
PECR	908.5 [333.2;2297.0]	1084.0 [346.6;3643.9]	1238.3 [526.7;2538.0]	0.202	0.192
PLA2R	13.9 [7.6;24.0]	14.7 [8.0;20.7]	15.2 [9.5;25.8]	0.714	0.296
PPIA	873.5 [434.0;1425.0]	948.2 [496.2;2041.1]	714.6 [378.9;1624.4]	0.338	0.636
PRKCH	1249.0 [791.0;2295.0]	1040.4 [648.8;1518.3]	844.4 [578.2;1536.9]	0.183	0.02
PRKCZ	2748.0 [1433.0;7042.0]	3902.2 [1638.2;9373.5]	4292.9 [1758.5;8799.2]	0.187	0.161
PTPRN	695.5 [334.8;1647.8]	578.2 [303.9;1010.3]	508.4 [300.5;1071.7]	0.165	0.233
REG3A	204.9 [152.0;396.6]	196.0 [126.0;310.4]	183.9 [130.9;281.1]	0.256	0.119
TNFA	257.5 [159.2;491.8]	245.0 [153.8;366.5]	220.1 [148.8;353.3]	0.351	0.162
TUBA1B	249.0 [178.4;665.7]	396.0 [168.2;781.5]	255.1 [126.1;520.8]	0.433	0.417
VM	620.0 [396.5;1274.8]	722.0 [349.5;1900.4]	614.2 [261.1;1317.1]	0.66	0.394

Median and interquartile ranges are presented. P values were from Mann-Whitney U test.

Supplemental Table 3. The proportion of kidney transplant recipients with the upper quartile ranges (≥ 75 percentile) of according to antibody levels.

Antigen for non-HLA antibody	ABMR group (N = 68)	No rejection controls (N = 83)	TCMR group (N = 67)	P (ABMR vs. no rejection)	P (ABMR vs. TCMR)
Collagen I	34 (50.0%)	8 (11.9%)	13 (15.7%)	< 0.001	< 0.001
Collagen II	19 (27.9%)	10 (14.9%)	26 (31.3%)	0.103	0.784
Collagen III	33 (48.5%)	8 (11.9%)	14 (16.9%)	< 0.001	< 0.001
Collagen IV	27 (40.3%)	6 (9.0%)	22 (26.5%)	< 0.001	0.106
Collagen V	19 (27.9%)	11 (16.4%)	25 (30.1%)	0.161	0.91
AGRIN	19 (27.9%)	17 (25.4%)	19 (22.9%)	0.887	0.601
AGT	13 (19.1%)	15 (22.4%)	27 (32.5%)	0.798	0.094
ARHGDIB	17 (25.0%)	10 (14.9%)	28 (33.7%)	0.212	0.323
AURKA	17 (25.0%)	12 (17.9%)	26 (31.3%)	0.428	0.499
CD36	11 (16.2%)	15 (22.4%)	29 (34.9%)	0.486	0.016
CHAF1B	20 (29.4%)	14 (20.9%)	21 (25.3%)	0.346	0.703
CXCL10	21 (30.9%)	10 (14.9%)	24 (28.9%)	0.046	0.933
CXCL11	25 (36.8%)	14 (20.9%)	16 (19.3%)	0.065	0.026
CXCL9	16 (23.5%)	8 (11.9%)	31 (37.3%)	0.125	0.099
EIF2A	17 (25.0%)	16 (23.9%)	22 (26.5%)	> 0.999	0.981
ENO1	16 (23.5%)	23 (34.3%)	16 (19.3%)	0.232	0.663
Fibronectin	19 (27.9%)	13 (19.4%)	23 (27.7%)	0.335	> 0.999
FLRT2	17 (25.0%)	11 (16.4%)	27 (32.5%)	0.309	0.405
GAPDH	19 (27.9%)	14 (20.9%)	22 (26.5%)	0.452	0.989
GDNF	19 (27.9%)	8 (11.9%)	28 (33.7%)	0.035	0.556
GSTT1	14 (20.6%)	20 (29.9%)	21 (25.3%)	0.298	0.625
HNRNPK	18 (26.5%)	10 (14.9%)	27 (32.5%)	0.149	0.528
IFIH1	17 (25.0%)	12 (17.9%)	26 (31.3%)	0.428	0.499
IFNG	22 (32.4%)	9 (13.4%)	24 (28.9%)	0.016	0.78
LG3	18 (26.5%)	14 (20.9%)	23 (27.7%)	0.576	> 0.999
LMNA	14 (20.6%)	16 (23.9%)	25 (30.1%)	0.8	0.252
LMNB	20 (29.4%)	15 (22.4%)	20 (24.1%)	0.463	0.582
MYOSIN	22 (32.4%)	15 (22.4%)	18 (21.7%)	0.269	0.196
NCL	23 (33.8%)	9 (13.4%)	23 (27.7%)	0.01	0.526
PECR	13 (19.1%)	16 (23.9%)	26 (31.3%)	0.643	0.129
PLA2R	17 (25.0%)	21 (31.3%)	17 (20.5%)	0.53	0.642
PPIA	13 (19.1%)	16 (23.9%)	26 (31.3%)	0.643	0.129
PRKCH	22 (32.4%)	15 (22.4%)	18 (21.7%)	0.269	0.196
PRKCZ	11 (16.2%)	19 (28.4%)	25 (30.1%)	0.135	0.07
PTPRN	23 (33.8%)	15 (22.4%)	17 (20.5%)	0.199	0.096
REG3A	23 (33.8%)	11 (16.4%)	21 (25.3%)	0.033	0.334
TNFA	23 (33.8%)	14 (20.9%)	18 (21.7%)	0.136	0.138
TUBA1B	16 (23.5%)	12 (17.9%)	27 (32.5%)	0.553	0.299
VM	13 (19.1%)	16 (23.9%)	26 (31.3%)	0.643	0.129

Numbers and percentages are presented. P values were from the chi-squared tests.

Supplemental Table 4. Clinical characteristics of the ABMR cases according to presence of high (≥ 75 percentile) anti-collagen type I level.

	High anti-collagen type I level (N = 34)	Low anti-collagen type I level (N = 34)	P value
Age at diagnosis (years)	50.0 [45.0;60.0]	45.5 [30.0;58.0]	0.13
Sex			0.63
Female	17 (50.0%)	14 (41.2%)	
Male	17 (50.0%)	20 (58.8%)	
Relation			0.06
Living related	8 (23.5%)	9 (26.5%)	
Living unrelated	8 (23.5%)	16 (47.1%)	
Cadaveric	18 (52.9%)	9 (26.5%)	
Sensitization-related status			
Number of HLA mismatch	4.0 [3.0; 5.0]	4.0 [3.0; 5.0]	0.75
ABO incompatibility	2 (6.2%)	12 (35.3%)	0.01
Presence of HLA-DSAs at diagnosis	17 (50.0%)	14 (41.2%)	0.63
PRA class I screening (%)	0.0 [0.0;24.0]	8.0 [0.0;44.0]	0.41
PRA class II screening (%)	27.0 [0.0;81.0]	0.0 [0.0;43.0]	0.10
Maintenance immunosuppression			
Calcineurin inhibitors			
Tacrolimus	27 (79.4%)	30 (88.2%)	0.51
Cyclosporine	5 (14.7%)	3 (8.8%)	0.71
Mycophenolic acid	22 (64.7%)	24 (70.6%)	0.80
Steroid	34 (100.0%)	33 (97.1%)	> 0.99
Desensitization	7 (21.2%)	12 (38.7%)	0.21
Laboratory findings			
Systolic BP (mmHg)	133.0 [121.0;141.0]	135.0 [124.0;145.0]	0.69
Diastolic BP (mmHg)	82.5 [73.0;90.0]	85.0 [78.0;92.0]	0.27
Serum protein (g/dL)	0.0 [0.0; 0.0]	0.0 [0.0;61.3]	0.23
Serum albumin (g/dL)	167.7 [155.1;171.4]	163.7 [157.1;172.7]	0.85
BUN (mg/dL)	6.2 [5.5; 7.0]	6.1 [5.4; 6.7]	0.39
Creatinine (mg/dL)	3.7 [3.2; 4.1]	3.6 [3.3; 4.0]	0.65
Proteinuria (g/g or g/day)	30.5 [25.0;42.0]	34.0 [19.0;45.0]	0.64

Supplemental Table 5. Clinical characteristics of the ABMR cases according to presence of high (≥ 75 percentile) anti-collagen type III level.

	ABMR with high anti-collagen type III level (N = 33)	ABMR with low anti-collagen type III level (N = 35)	P value
Age at diagnosis (years)	50.0 [45.0;58.0]	46.0 [30.0;58.0]	0.23
Sex			> 0.99
Female	15 (45.5%)	16 (45.7%)	
Male	18 (54.5%)	19 (54.3%)	
Relation			0.03
Living related	8 (24.2%)	9 (25.7%)	
Living unrelated	7 (21.2%)	17 (48.6%)	
Cadaveric	18 (54.5%)	9 (25.7%)	
Sensitization-related status			
Number of HLA mismatch	4.0 [3.0; 5.0]	4.0 [3.0; 5.0]	0.72
ABO incompatibility	2 (6.5%)	12 (34.3%)	0.01
Presence of HLA-DSAs at diagnosis	17 (51.5%)	14 (40.0%)	0.48
PRA class I screening (%)	1.0 [0.0;27.0]	1.0 [0.0;41.5]	0.88
PRA class II screening (%)	32.5 [0.0;82.0]	0.0 [0.0;40.5]	0.03
Maintenance immunosuppression			
Calcineurin inhibitors			
Tacrolimus	26 (78.8%)	31 (88.6%)	0.44
Cyclosporine	5 (15.2%)	3 (8.6%)	0.64
Mycophenolic acid	21 (63.6%)	25 (71.4%)	0.67
Steroid	33 (100.0%)	34 (97.1%)	> 0.99
Desensitization	6 (18.8%)	13 (40.6%)	0.10
Laboratory findings			
Systolic BP (mmHg)	134.0 [120.0;140.0]	135.0 [124.0;143.0]	0.60
Diastolic BP (mmHg)	83.0 [73.0;90.0]	85.0 [79.0;94.0]	0.18
Serum protein (g/dL)	6.3 [5.5; 7.0]	6.0 [5.4; 6.7]	0.27
Serum albumin (g/dL)	3.7 [3.3; 4.1]	3.6 [3.2; 4.0]	0.47
BUN (mg/dL)	30.0 [20.0;51.0]	33.5 [19.0;44.0]	0.63
Creatinine (mg/dL)	1.8 [1.2; 3.3]	1.6 [1.0; 2.6]	0.39
Proteinuria (g/g or g/day)	0.8 [0.4; 1.6]	0.8 [0.3; 2.0]	0.83

Supplemental Table 6. Non-HLA antibody levels according to presence of HLA-DSAs

Antigen for non-HLA antibody	ABMR cases without HLA-DSAs (N = 37)	ABMR cases with HLA-DSAs (N = 31)	P
Collagen I	60.5 [37.0;184.0]	92.3 [25.2;147.9]	0.777
Collagen II	7.4 [3.2;13.1]	3.3 [0.0; 8.2]	0.045
Collagen III	80.4 [55.2;213.5]	106.6 [48.2;248.0]	0.73
Collagen IV	45.4 [31.4;64.0]	29.5 [18.2;57.5]	0.111
Collagen V	41.7 [27.6;68.2]	34.5 [18.1;56.2]	0.319
AGRIN	63.0 [29.0;98.0]	48.0 [31.5;151.9]	0.975
AGT	392.3 [250.0;866.0]	624.0 [321.5;1101.2]	0.185
ARHGDIB	397.0 [298.5;694.0]	442.8 [314.9;681.2]	0.389
AURKA	1129.0 [716.0;1774.0]	1173.7 [652.5;2113.5]	0.807
CD36	35.9 [11.7;99.0]	51.0 [20.4;117.3]	0.272
CHAF1B	2664.0 [1038.0;4192.8]	2696.7 [1177.0;3605.8]	0.616
CXCL10	168.4 [121.0;281.6]	148.8 [120.7;239.0]	0.567
CXCL11	235.0 [163.0;494.9]	202.3 [155.1;392.5]	0.209
CXCL9	65.0 [48.9;107.9]	48.0 [37.4;98.9]	0.198
EIF2A	682.0 [351.0;981.0]	770.4 [244.9;2741.6]	0.874
ENO1	1771.0 [1026.0;3845.0]	1465.0 [845.3;2899.5]	0.426
Fibronectin	25.5 [19.3;32.6]	18.5 [11.9;23.0]	0.01
FLRT2	460.4 [291.0;661.4]	452.8 [333.2;720.2]	0.631
GAPDH	183.0 [149.0;231.0]	198.3 [148.3;277.7]	0.763
GDNF	342.0 [205.0;615.0]	301.5 [162.0;424.5]	0.431
GSTT1	964.0 [674.0;1385.8]	780.0 [503.5;1313.4]	0.197
HNRNPK	479.0 [390.0;738.0]	507.0 [399.4;994.6]	0.396
IFIH1	539.0 [224.0;842.0]	679.3 [215.9;2047.8]	0.268
IFNG	549.7 [272.6;863.0]	544.0 [316.8;766.5]	0.99
LG3	304.8 [201.0;583.0]	385.0 [290.0;1447.7]	0.089
LMNA	1672.0 [1009.5;2690.0]	1401.0 [917.3;2036.0]	0.524
LMNB	690.0 [356.0;1041.0]	826.9 [549.3;1474.2]	0.108
MYOSIN	2921.0 [1866.0;5417.0]	2655.8 [1982.4;5220.5]	0.75
NCL	130.0 [82.0;326.0]	145.0 [75.6;305.2]	0.985
PECR	769.0 [306.0;1488.8]	1137.0 [645.6;2474.8]	0.155
PLA2R	13.0 [8.0;21.0]	19.0 [7.7;27.6]	0.343
PPIA	858.0 [428.0;1279.0]	1024.3 [470.0;1553.1]	0.557
PRKCH	1140.0 [789.0;2131.0]	1315.7 [820.5;2529.0]	0.81
PRKCZ	4144.9 [1648.0;7560.4]	2374.0 [1233.0;4777.5]	0.083
PTPRN	585.0 [320.0;903.3]	1351.0 [430.9;1926.0]	0.054
REG3A	200.0 [157.0;417.0]	224.9 [124.4;365.1]	0.498
TNFA	244.2 [156.4;511.0]	321.0 [181.0;477.4]	0.805
TUBA1B	289.5 [143.0;604.0]	244.0 [194.0;716.4]	0.514
VM	607.0 [348.0;1234.0]	636.0 [409.2;1376.2]	0.47

Median and interquartile ranges are presented. P values were from Mann-Whitney U test.

Supplemental Table 7. Non-HLA antibody levels according to presence of chronic-active AMR.

Antigen for non-HLA antibody	ABMR cases without chronic-active lesion (N = 49)	ABMR cases with chronic-active lesions \(N = 19)	P
Collagen I	80.8 [31.5;188.2]	60.5 [30.5;118.3]	0.53
Collagen II	5.0 [1.4;12.2]	6.3 [2.6;10.4]	0.716
Collagen III	102.9 [55.7;255.3]	78.6 [43.4;149.4]	0.389
Collagen IV	41.2 [26.1;64.0]	31.2 [11.0;52.9]	0.261
Collagen V	41.7 [23.6;62.5]	41.2 [21.6;54.4]	0.613
AGRIN	71.0 [31.0;127.4]	44.1 [25.1;71.5]	0.211
AGT	502.0 [305.0;911.0]	523.7 [245.6;903.4]	0.745
ARHGDIB	435.5 [327.0;828.0]	349.0 [277.2;546.7]	0.116
AURKA	1303.0 [716.0;2044.3]	1104.7 [737.5;1612.4]	0.37
CD36	40.0 [11.6;101.9]	43.3 [16.9;117.3]	0.622
CHAF1B	3132.0 [1393.1;4207.0]	1664.8 [1102.6;2911.6]	0.069
CXCL10	177.0 [134.0;307.0]	132.0 [93.4;172.7]	0.011
CXCL11	268.5 [182.0;478.0]	154.1 [125.5;285.1]	0.005
CXCL9	62.0 [44.0;97.1]	52.0 [41.3;153.9]	0.854
EIF2A	706.0 [343.0;2178.0]	542.3 [254.4;1479.6]	0.551
ENO1	2104.0 [1042.0;3956.0]	1167.3 [739.5;1759.2]	0.053
Fibronectin	23.5 [17.1;30.6]	18.8 [13.6;22.7]	0.151
FLRT2	461.0 [307.9;658.0]	452.8 [341.4;722.3]	0.785
GAPDH	195.0 [149.0;250.3]	182.8 [140.6;207.5]	0.682
GDNF	386.0 [220.0;645.0]	187.0 [131.4;267.9]	0.004
GSTT1	971.0 [593.9;1621.0]	780.0 [489.8;929.0]	0.098
HNRNPK	507.0 [440.2;957.0]	456.7 [366.9;616.5]	0.063
IFIH1	597.0 [289.7;1965.0]	547.9 [160.6;1113.4]	0.575
IFNG	570.0 [382.0;822.0]	306.2 [183.0;801.6]	0.063
LG3	452.0 [251.3;1378.4]	300.0 [214.6;400.5]	0.073
LMNA	1514.5 [987.0;2407.1]	1668.2 [956.5;2687.4]	0.714
LMNB	622.0 [395.0;1225.0]	950.9 [652.7;1473.3]	0.157
MYOSIN	3886.0 [2201.0;5868.0]	2308.0 [1511.2;2622.3]	0.003
NCL	155.0 [90.0;362.0]	83.9 [58.5;153.0]	0.013
PECR	971.0 [441.0;2281.0]	726.0 [206.2;1900.8]	0.42
PLA2R	15.0 [10.0;25.0]	10.3 [6.1;17.5]	0.073
PPIA	893.9 [508.0;1435.0]	679.1 [326.1;1183.9]	0.156
PRKCH	1283.0 [836.3;2131.0]	1215.0 [709.0;2369.2]	0.956
PRKCZ	3745.2 [1725.0;7219.0]	1403.9 [843.8;2917.9]	0.002
PTPRN	731.0 [346.0;1725.0]	513.6 [266.8;714.3]	0.037
REG3A	248.0 [157.0;400.0]	173.0 [130.3;234.4]	0.076
TNFA	339.0 [189.0;558.0]	155.8 [121.7;253.6]	0.002
TUBA1B	328.0 [188.6;682.8]	241.3 [130.7;590.4]	0.404
VM	636.0 [397.0;1234.0]	513.2 [397.4;1401.6]	0.957

Median and interquartile ranges are presented. P values were from Mann-Whitney U test.
Chronic active lesions were diagnosed when morphologic evidence of chronic tissue injury such as glomerular double contours and/or peritubular basement membrane multilayering and/or interstitial fibrosis/tubular atrophy and/or fibrous intimal thickening in arteries was present.

Supplemental Table 8. Non-HLA antibody levels according to coexistence of TCMR or calcineurin inhibitor toxicity.

Non-HLA antibodies	ABMR cases without other findings (N = 27)	ABMR cases with coexisting TCMR or calcineurin inhibitor toxicity (N = 41)	P
EIF2A	846.2 [594.8;3289.2]	483.1 [258.4;1105.4]	0.022
Collagen II	8.8 [3.4;28.8]	3.6 [1.6; 8.2]	0.024
Fibronectin	28.6 [17.2;36.2]	20.5 [13.3;25.7]	0.024
CXCL9	77.0 [53.0;133.0]	52.0 [40.4;76.0]	0.064
GDNF	409.0 [244.7;785.9]	220.5 [172.0;443.0]	0.066
IFNG	712.0 [360.0;1089.2]	501.0 [271.6;641.0]	0.087
PRKCH	1464.8 [878.8;3001.3]	1117.0 [694.0;1720.0]	0.12
HNRNPK	657.0 [422.5;985.9]	477.8 [386.0;636.0]	0.128
IFIH1	842.0 [404.8;2329.9]	428.7 [223.0;799.2]	0.136
Collagen V	43.9 [27.1;67.6]	36.4 [19.2;55.3]	0.136
CXCL11	277.0 [177.6;502.1]	195.0 [156.0;413.0]	0.168
AURKA	1516.0 [876.0;2197.7]	1104.7 [586.0;1819.0]	0.194
FLRT2	512.0 [331.5;813.5]	397.0 [307.9;629.8]	0.201
MYOSIN	3857.0 [2002.7;5699.0]	2628.0 [1856.8;4386.0]	0.207
ENO1	2104.0 [1264.1;3197.9]	1465.0 [780.0;3244.0]	0.216
PRKCZ	5276.5 [1634.0;7210.5]	2062.2 [1403.9;4965.0]	0.226
CD36	45.0 [23.4;127.0]	39.0 [7.5;89.7]	0.289
CXCL10	201.0 [115.0;305.1]	147.0 [122.0;242.0]	0.298
TUBA1B	361.0 [191.8;761.4]	244.0 [160.0;434.0]	0.347
AGT	562.0 [315.0;1153.4]	413.0 [284.2;808.2]	0.432
ARHGDIB	428.0 [337.1;688.3]	416.2 [282.0;679.8]	0.467
Collagen IV	44.8 [25.0;95.9]	33.8 [23.3;59.6]	0.47
VM	696.8 [415.4;1388.4]	582.6 [385.0;1005.2]	0.501
AGRIN	44.1 [27.0;116.2]	56.1 [31.0;98.0]	0.527
LMNA	1672.0 [1022.3;2280.9]	1374.9 [931.0;2690.0]	0.533
LG3	385.0 [290.0;980.7]	332.0 [225.3;888.8]	0.539
PTPRN	718.0 [367.6;1691.8]	678.3 [329.0;1527.4]	0.654
NCL	135.0 [81.5;327.9]	129.0 [77.2;312.3]	0.712
PLA2R	15.0 [8.0;22.4]	13.0 [7.2;25.0]	0.821
GSTT1	868.0 [599.0;1359.1]	859.7 [558.0;1543.6]	0.842
LMNB	790.0 [376.0;1281.1]	692.0 [490.6;1225.0]	0.846
TNFA	286.0 [169.7;490.3]	244.2 [162.0;486.0]	0.846
PPIA	901.4 [429.5;1449.5]	852.0 [471.0;1324.0]	0.881
Collagen I	80.8 [24.4;185.1]	70.9 [32.2;152.5]	0.91
CHAF1B	2106.0 [900.0;3931.4]	2696.7 [1393.1;3793.0]	0.911
GAPDH	195.0 [161.5;232.6]	183.0 [132.6;288.5]	0.96
Collagen III	80.4 [54.2;257.9]	102.9 [54.1;210.4]	0.96
PECR	871.6 [302.5;2226.2]	937.0 [375.9;2281.0]	0.975
REG3A	225.2 [152.5;383.7]	204.0 [152.0;400.0]	> 0.999

Median and interquartile ranges are presented. P values were from Mann-Whitney U test.

Supplemental Table 9. Association between ischemic time and anti-collagen I and III level (MFI) within the antibody mediated rejection group.

Exposure and outcome variable	Univariable model		Multivariable model	
	beta (standard error)	P	adjusted beta (standard error)	P
Collagen I				
Cold ischemic time (per minute)	2.776 (0.980)	0.007	2.706 (1.080)	0.018
Warm ischemic time (per minute)	-0.755 (9.463)	0.937	-6.150 (10.248)	0.552
Collagen III				
Cold ischemic time (per minute)	2.130 (1.051)	0.050	2.139 (1.152)	0.073
Warm ischemic time (per minute)	-1.133 (9.416)	0.905	-5.538 (10.339)	0.595

Multivariable model was adjusted for age, serum creatinine, presence of donor specific antibody at the time of diagnosis for antibody mediated rejection, and the duration from transplantation to diagnosis of the antibody-mediated rejection.

Supplemental Table 10. Banff classification according to levels of anti-collagen type I or type III antibodies.

Banff scores	Anti-collagen type I antibody level ≥ 75 percentile (N = 34)	Anti-collagen type I antibody level < 75 percentile (N = 34)	p	Anti-collagen type III antibody level ≥ 75 percentile (N = 33)	Anti-collagen type III antibody level < 75 percentile (N = 35)	p
t			0.52	0		0.272
0	12 (35.3%)	18 (52.9%)		11 (33.3%)	19 (54.3%)	
1	15 (44.1%)	11 (32.4%)		16 (48.5%)	10 (28.6%)	
2	5 (14.7%)	4 (11.8%)		4 (12.1%)	5 (14.3%)	
3	2 (5.9%)	1 (2.9%)		2 (6.1%)	1 (2.9%)	
v			0.353			0.323
0	24 (70.6%)	26 (76.5%)		23 (69.7%)	27 (77.1%)	
1	8 (23.5%)	8 (23.5%)		8 (24.2%)	8 (22.9%)	
2	2 (5.9%)	0 (0.0%)		2 (6.1%)	0 (0.0%)	
i			0.041			0.271
0	3 (8.8%)	11 (32.4%)		4 (12.1%)	10 (28.6%)	
1	23 (67.6%)	13 (38.2%)		21 (63.6%)	15 (42.9%)	
2	6 (17.6%)	9 (26.5%)		7 (21.2%)	8 (22.9%)	
3	2 (5.9%)	1 (2.9%)		1 (3.0%)	2 (5.7%)	
g			0.389			0.523
0	13 (38.2%)	16 (48.5%)		13 (39.4%)	16 (47.1%)	
1	16 (47.1%)	10 (30.3%)		15 (45.5%)	11 (32.4%)	
2	3 (8.8%)	6 (18.2%)		3 (9.1%)	6 (17.6%)	
3	2 (5.9%)	1 (3.0%)		2 (6.1%)	1 (2.9%)	
ci			0.463			0.714
0	21 (61.8%)	25 (73.5%)		22 (66.7%)	24 (68.6%)	
1	11 (32.4%)	7 (20.6%)		9 (27.3%)	9 (25.7%)	
2	1 (2.9%)	2 (5.9%)		1 (3.0%)	2 (5.7%)	
3	1 (2.9%)	0 (0.0%)		1 (3.0%)	0 (0.0%)	
ct			0.158			0.587
0	23 (67.6%)	22 (64.7%)		22 (66.7%)	23 (65.7%)	
1	5 (14.7%)	10 (29.4%)		6 (18.2%)	9 (25.7%)	
2	6 (17.6%)	2 (5.9%)		5 (15.2%)	3 (8.6%)	
cg			0.137			0.138
0	24 (70.6%)	20 (58.8%)		22 (66.7%)	22 (62.9%)	
1	5 (14.7%)	5 (14.7%)		6 (18.2%)	4 (11.4%)	
2	1 (2.9%)	7 (20.6%)		1 (3.0%)	7 (20.0%)	
3	4 (11.8%)	2 (5.9%)		4 (12.1%)	2 (5.7%)	
mm			0.466			0.493
0	29 (85.3%)	26 (78.8%)		28 (84.8%)	27 (79.4%)	
1	4 (11.8%)	6 (18.2%)		4 (12.1%)	6 (17.6%)	
2	0 (0.0%)	1 (3.0%)		0 (0.0%)	1 (2.9%)	
3	1 (2.9%)	0 (0.0%)		1 (3.0%)	0 (0.0%)	
cv			0.994			0.999
0	22 (64.7%)	21 (61.8%)		21 (63.6%)	22 (62.9%)	
1	8 (23.5%)	9 (26.5%)		8 (24.2%)	9 (25.7%)	
2	3 (8.8%)	3 (8.8%)		3 (9.1%)	3 (8.6%)	
3	1 (2.9%)	1 (2.9%)		1 (3.0%)	1 (2.9%)	
ah			0.436			0.766
0	30 (88.2%)	25 (75.8%)		27 (81.8%)	28 (82.4%)	
1	2 (5.9%)	5 (15.2%)		4 (12.1%)	3 (8.8%)	
2	2 (5.9%)	2 (6.1%)		2 (6.1%)	2 (5.9%)	
3	0 (0.0%)	1 (3.0%)		0 (0.0%)	1 (2.9%)	
ptc			0.04			0.047
0	2 (5.9%)	11 (32.4%)		2 (6.1%)	11 (31.4%)	
1	16 (47.1%)	13 (38.2%)		15 (45.5%)	14 (40.0%)	
2	11 (32.4%)	8 (23.5%)		11 (33.3%)	8 (22.9%)	
3	5 (14.7%)	2 (5.9%)		5 (15.2%)	2 (5.7%)	

There was one case with missing information for some Banff lesions (e.g. g, mm).

Supplemental Table 11. Comparison of the anti-collagen type I and type III antibody levels before and after transplantation in 47 ABMR cases.

Non-HLA antibodies	Before transplantation (N = 47)	At the time of ABMR diagnosis (N = 47)	P (Paired t-test for continuous value)
Collagen I			
Continuous (MFI)	43.2 [12.3; 110.0]	49.9 [21.4; 112.0]	0.115
Categorical (≥ 75 percentile of the main analysis)	16 (34.0%)	20 (42.6%)	0.572
Collagen III			
Continuous (MFI)	75.3 [47.5; 167.4]	69.2 [29.8; 161.1]	0.413
Categorical (≥ 75 percentile of the main analysis)	18 (38.3%)	19 (40.4%)	0.157

Supplemental Table 12. Risk of death-censored graft failure of the subgroups.

Subgroup	N of DCGF/N of total	Univariable model		Multivariable model		Reference group changed to TCMR, multivariable model	
		HR (95% CI)	P	Adjusted HR (95% CI)	P	Adjusted HR (95% CI)	P
No rejection	1/82	Reference		Reference		0.42 (0.04-4.09)	0.45
TCMR	3/67	3.01 (0.31-29.04)	0.34	2.41 (0.24-23.77)	0.45	Reference	
ABMR with HLA-DSA	6/31	20.94 (2.52-174.32)	0.005	11.45 (1.24-105.32)	0.03	4.75 (1.07-21.01)	0.04
ABMR without HLA-DSA	7/37	17.74 (2.18-144.28)	0.007	11.99 (1.29-111.40)	0.03	4.97 (1.12-22.14)	0.04

HR = hazard ratio, CI = confidence interval, TCMR = T-cell mediated rejection, ABMR = antibody-mediated rejection, HLA-DSA = donor-specific human-leukocyte antigen antibody

Multivariable model was adjusted for age, serum creatinine, and duration from transplantation to serum acquisition (which was near biopsy)

Supplemental Figure 1. Correlation between anti-collagen type I and type III antibody level.

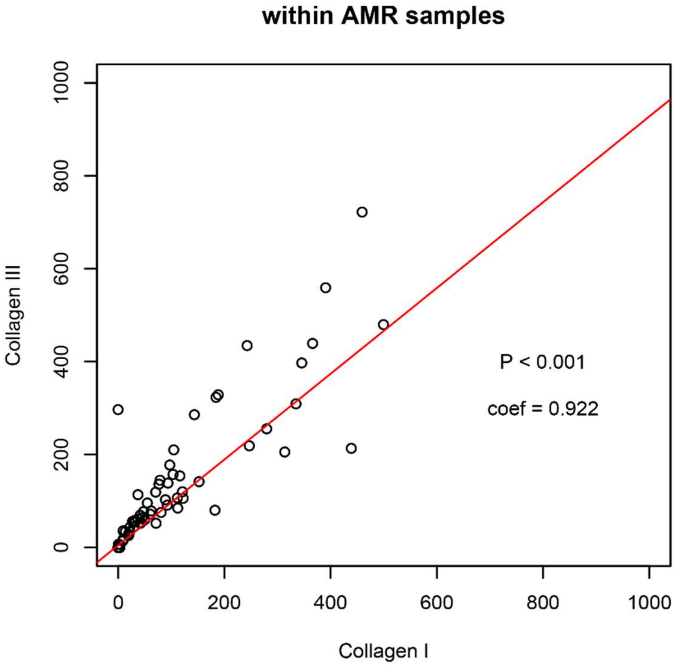

X-axis indicate the levels of anti-collagen I antibody level and the y-axis indicate the levels of anti-collagen III antibody level. The units were in mean fluorescence intensity. Coefficient was calculated from Pearson's correlation test.

Supplemental Figure 2. Investigation for the linearlity of the association between non-HLA antibody levels and odds for ABMR.

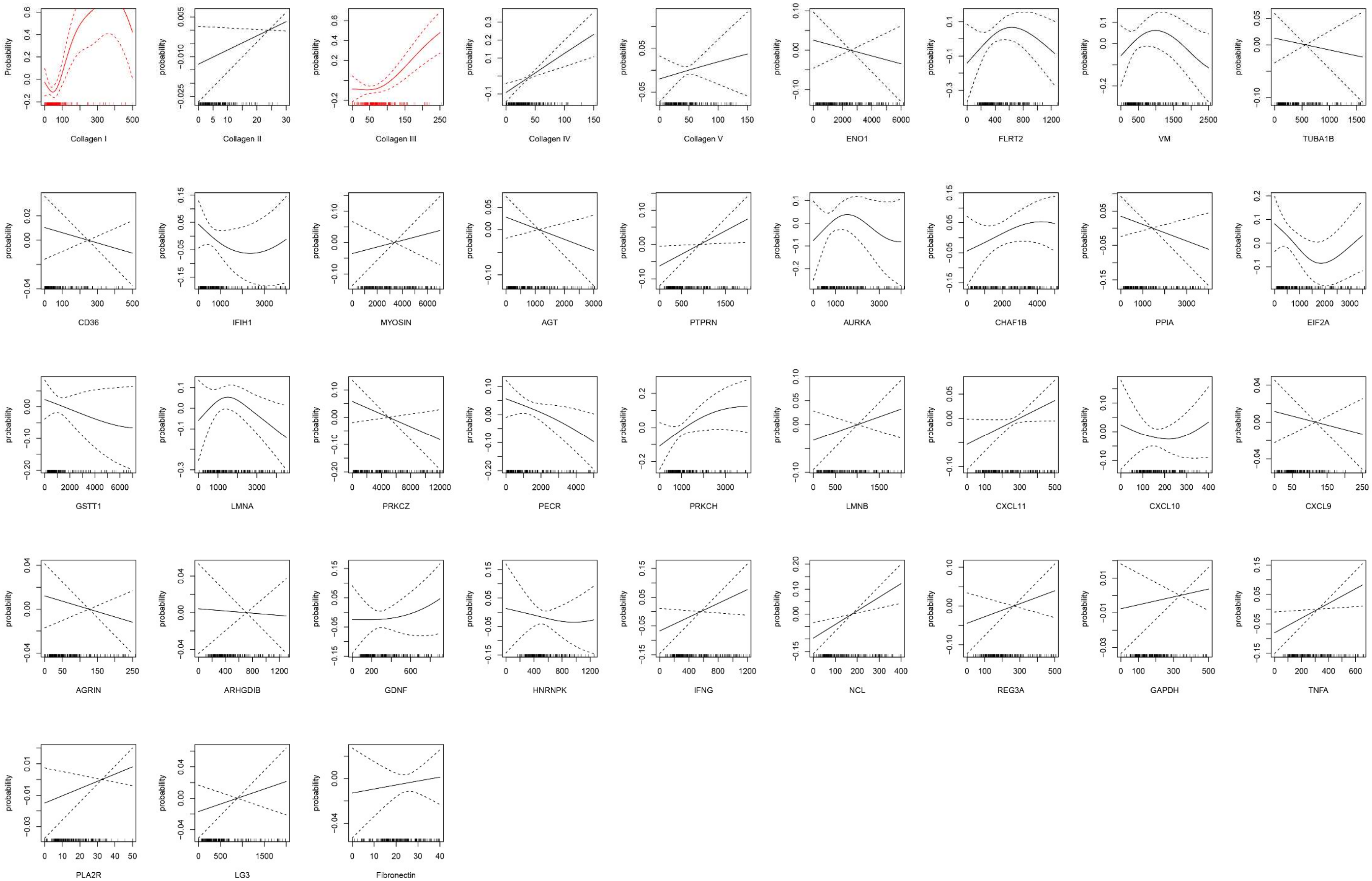

Generalized additive model was used to plot the graphs, and the predicted probability of ABMR among the study population was plotted according to each non-HLA antibody level.

Supplemental Figure 3. The correlation between anti-collagen type I or type III antibody titers and Banff lesions, including both ABMR and TCMR cases.

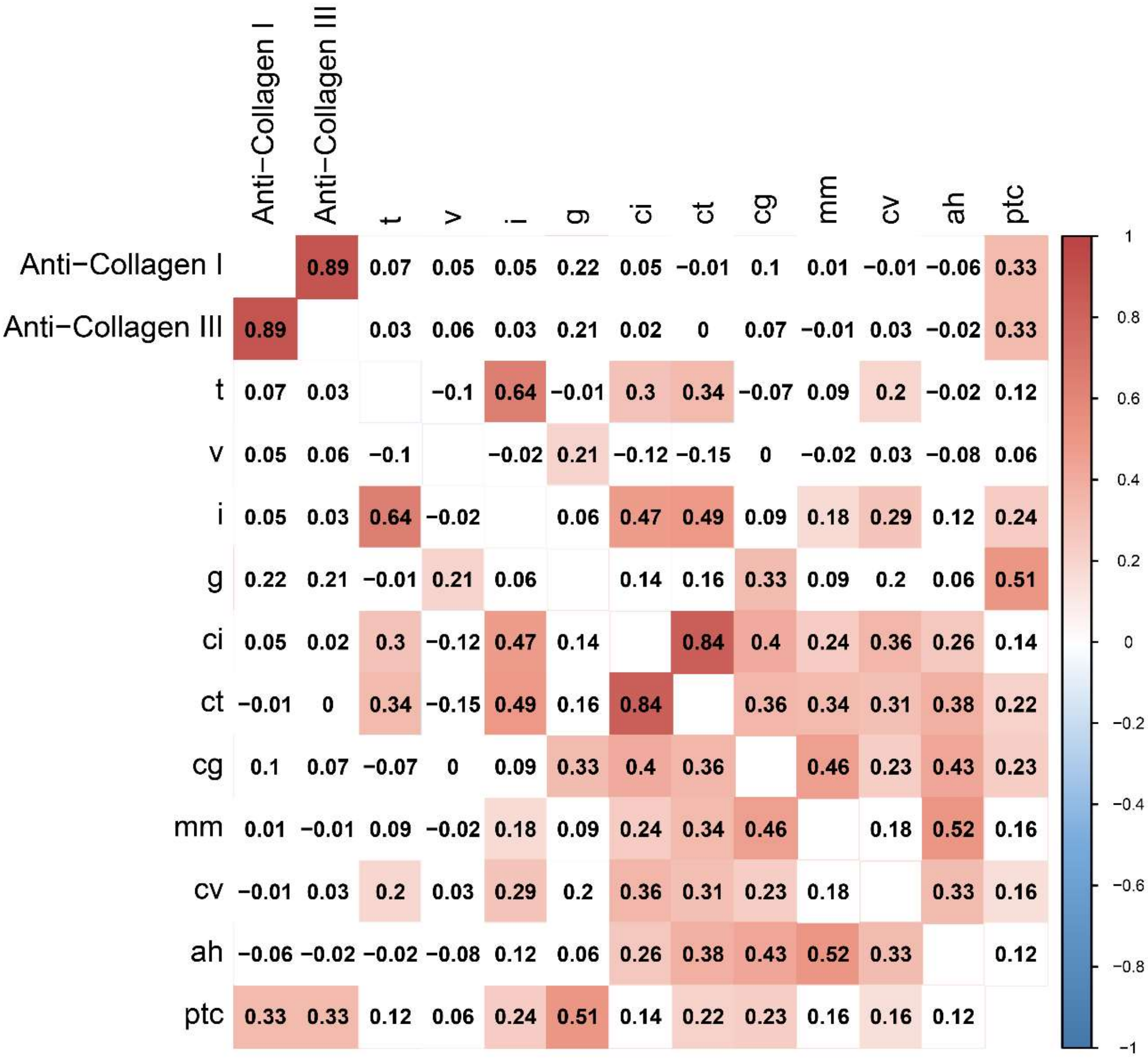

The correlation indices were calculated by the Spearman's method and appear as colors. Positive correlations are presented by red and darker color means a stronger correlation. Only the correlation that reached statistical significance ($P < 0.05$) were colored. All cases including TCMR and ABMR cases were included, except for one case excluded because some information of Banff classification was missing in the information.

Supplemental Figure 4. The correlation between the antibody values measured by ELISA method and the MFI titers measured by the bead-based Luminex method.

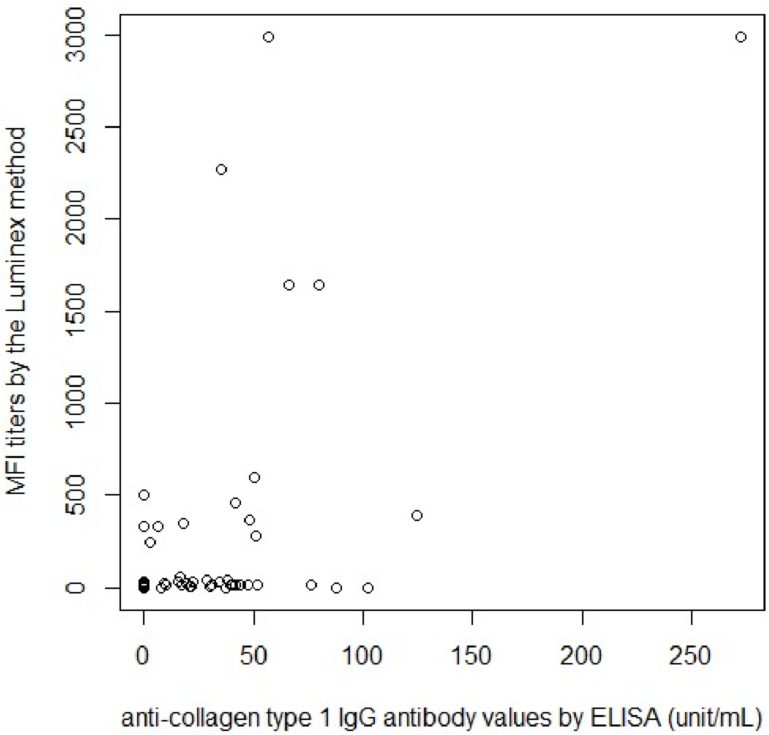

The OD-based anti-collagen I IgG antibody values measured by ELISA and the MFI titers measured by the Luminex method showed significant ($P < 0.001$) correlation with each other (Pearson R = 0.580, Supplemental Figure 4). In addition, the ABMR group showed average 44.9±57.5 unit/mL of anti-collagen I IgG antibody measured by ELISA, which was significantly ($P = 0.040$) higher that of the controls (20.4±21.6 unit/mL). Those with high (> 75 percentile) anti-collagen I antibody titers measured by the Luminex method were significantly associated with higher values (> 75 percentile) measured by ELISA (8/15, 53.3%), while those with the lower ranges of titers by the Luminex method also frequently showed low values by ELISA (33/39, 84.6%) ($P = 0.003$).
